# Epidemiology of proximal femur fractures in the young population of Qatar

**DOI:** 10.1007/s00590-023-03664-1

**Published:** 2023-08-07

**Authors:** Ashraf T. Hantouly, Asim AlBarazanji, Mohammed Al-Juboori, Mohanad Alebbini, Ahmad A. Toubasi, Asma Mohammed, Osama Alzobi, Ghalib Ahmed

**Affiliations:** 1 Department of Orthopedic Surgery, Surgical Specialty Center, Hamad Medical Corporation, Doha, Qatar; 2 University of Jordan, Amman, Jordan

**Keywords:** Hip fractures, Femur, Epidemiology, Qatar

## Abstract

**Purpose:**

To review the epidemiological characteristics of proximal femur fractures in the young population (< 60 years) of Qatar between 2017 and 2019.

**Methods:**

All patient treated for proximal femur fractures at Hamad General Hospital (HGH), a level one trauma center, were retrospectively reviewed between Jan 2017 and Dec 2019. All adults (18–60 years) with proximal femur fracture (femur head, femur neck, intertrochanteric and subtrochanteric fractures) were included with no restriction to the AO/OTA classification or fractures subtypes. Excluded cases were pathological fractures, cases with insufficient documentation or no radiographs.

**Results:**

A total of 203 patients with a mean age of 40.07 ± 11.76 years were included, of who 89.9% were males. The incidence of proximal femur fracture was 3.12/100,000/year. Fall from height (48.1%) followed by road traffic accidents (26.9%) were common cause of injury. The most common fracture type was intertrochanteric fracture (36.1%) followed by femur neck fractures (33.7%).

**Conclusion:**

This study provides the initial insights into the proximal femur fractures in the young population of Qatar. This is the first study to investigate of the epidemiology of such fractures in this particular patient group. Contrary to the existing literature on older age groups, the majority of the injuries were observed in males. Falls from height followed by road traffic accidents were the primary mechanisms leading to these fractures. Improved understanding of the profile of these injuries can aid in their prevention by implementing more effective safety measures.

## Introduction

The incidence of hip fractures has been increasing significantly over the last decades [1]. It is estimated that the annual incidence of hip fractures will rise up to 6 million fractures worldwide by the year 2050, 70% of which occurring in Asia, Latin America, the Middle East and Africa [2, 3].

Increased life expectancy and activity levels in older age, in addition to increased survival rates among younger trauma patients, explain the exponential rise in hip fracture rates [4]. As a result of this dramatic rise, the burden on health systems is expected to increase exponentially [5]. Up to 20 billion dollars are spent annually on hip fractures, making this type of fractures one of the most expensive fractures to treat [5]. Moreover, mortality rates of hip fractures are estimated to exceed 20% within one year and this percentage rises dramatically over time as the functional status of hip fracture patients deteriorates [6].

Hip fractures can be classified into intracapsular, such as femoral neck fractures, and extracapsular such as intertrochanteric and subtrochanteric fractures. Age distribution, mechanism of injury, management and outcomes differ between the different types and subtypes [1].

Qatar has one of the fastest growing economies in the world. The country’s rapid development has a substantial impact on its demographics and health indicators. More than 70% of the population are young expatriate male workers and approximately 83% of who falls within the age range of 15–65 years [7, 8]. This in turn, affects the incidence as well as the characteristics of hip fractures and their management.

As hip fractures have a significant impact on quality of life and increase morbidity and mortality, it is paramount to conduct an epidemiological study to investigate the incidence, characteristics and patterns of hip fractures in Qatar. Therefore, the aim of this retrospective epidemiological study is to review the epidemiological aspects of hip fractures of the young population (< 60 years) managed at Hamad General Hospital, the only level 1 trauma center in Qatar, between Jan 2017 and Dec 2019. We hypothesized that there is a higher incidence rate of hip fractures in young patients in Qatar as compared to other regions of the world.

## Methods

### Study design

Approval was obtained from the institutional review board at HGH, the only level one-trauma center in Qatar. All cases that were treated for proximal femur fractures at HGH, were retrospectively reviewed between Jan 2017 and Dec 2019.

### Eligibility criteria

The inclusion criteria were all adults (18–60 years) with proximal femur fracture (femur head, femur neck, intertrochanteric or subtrochanteric fractures) that were treated at HGH between Jan 2017 and Dec 2019. All fracture patterns were included with no restriction to the AO/OTA classification or fractures subtypes [9]. Excluded cases were pathological fractures, cases with insufficient documentation or no radiographs.

### Data source and collection

Data retrieved from the patients’ electronic medical files was assessed to identify eligible patients diagnosed with proximal femur fractures. The data items that were collected included: age, gender, comorbidities, bone marrow density, mechanism of injury, fracture classification, associated injuries, treatment modality, admission-to-surgery time, length of stay, and complications, reoperations and mortality.

### Fracture classification

Fractures were classified according to the anatomic location of the fracture (femur head, femoral neck, intertrochanteric and subtrochanteric fractures) and the AO/OTA classification, which proposes a uniform alphanumerical classification [9]. Subclassifications were also performed to femur neck fractures as per Pauwels’ angle and Garden’s classification [10, 11].

### Statistical analysis

The data analysis was done using IBM-SPSS v.25. Categorical variables were presented as counts and percentages whereas continuous variables were interpreted as mean, standard deviation and range. The difference in the characteristics according to the outcomes of the patients was done using Chi-square test and T-test for categorical and continuous variables, respectively. Any test with a *P*-value < 0.05 was considered significant.

## Results

**Fig. 1:**
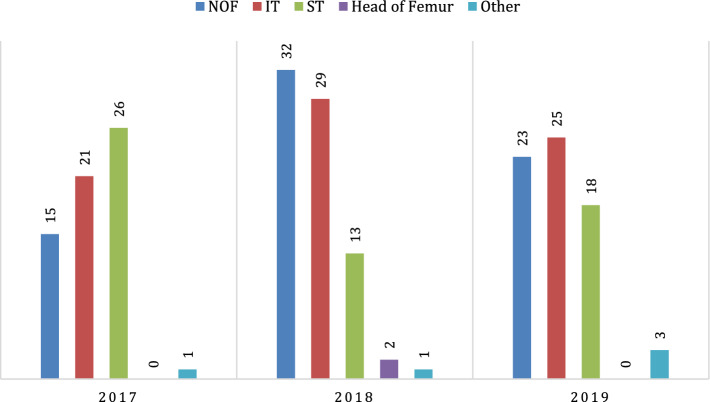
Proximal femur fractures at HGH between 2017 and 2019. *NOF* Neck of femur, *IT* Intertrochanteric, *ST* Subtrochanteric

The total number of the included patients between Jan 2017 and Dec 2019 was 203, four of whom had bilateral injuries and one patient had two fractures on two separate occasions. The overall incidence of PFF fractures between 2017 and 2019 was 3.12 per 100,000 population per year. The incidence in 2017 was 2.89 per 100,000 population per year (61 cases). Moreover, the incidence was 3.46 (75 cases) and 3.02 (67 cases) per 100,000 population per year in 2018 and 2019, respectively (Fig. 1). Table 1 demonstrates the demographic characteristics of the participants.

### Trends in age and gender

**Fig. 2:**
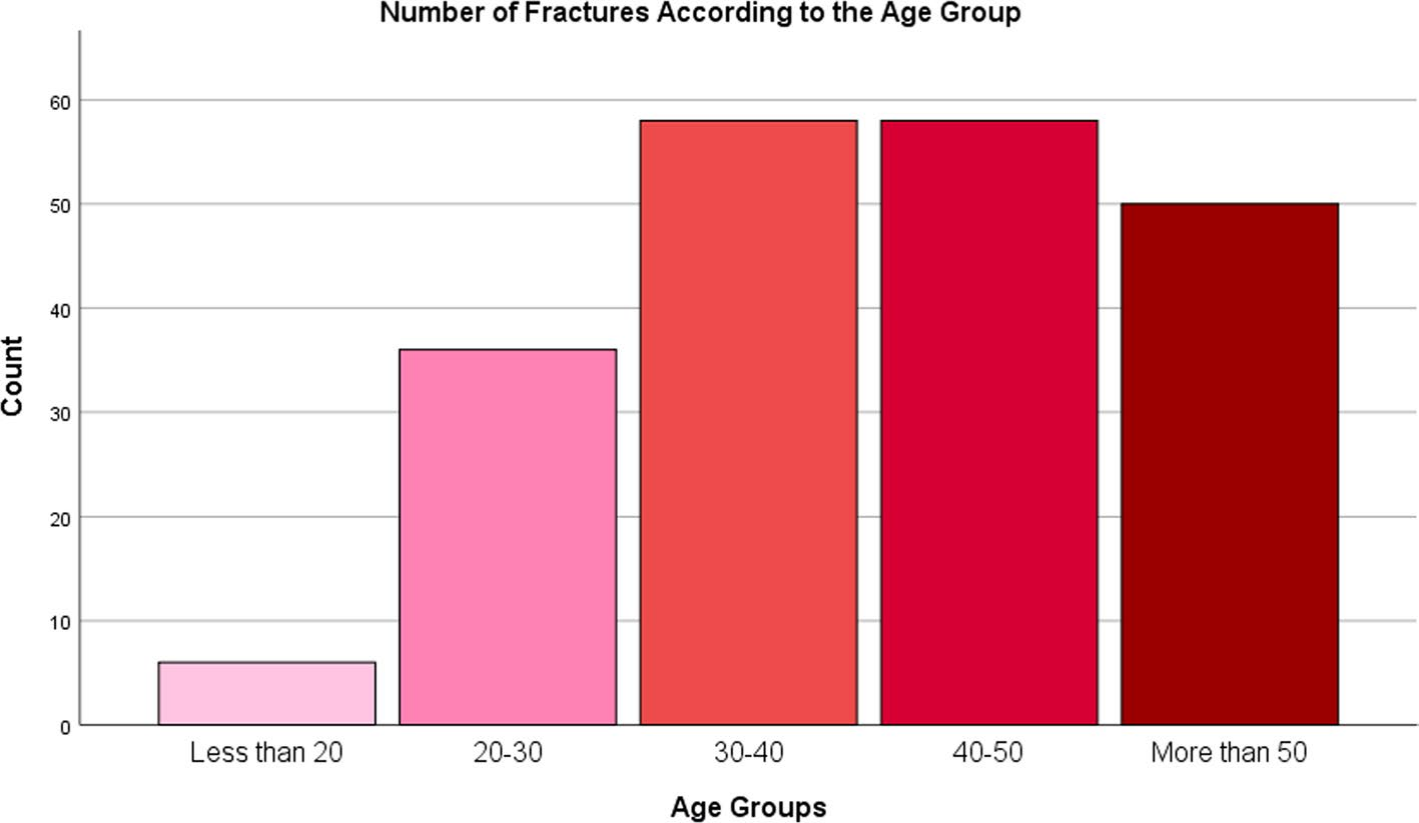
Number of fractures according to age group

The mean age of the included patients was 40.07 ± 11.76. The majority of the fractures were in the age group from 30 to 40 followed by the group from 40 to 50 while the lowest number of fractures was in the age group less than 20. Figure 2 shows the trends of fractures according to the age group. The majority of the patients were males (89.9%). The most common side of the injury was right side (53.1%) and 2.5% of the patients had bilateral injury.

### Mechanism of injury

**Fig. 3:**
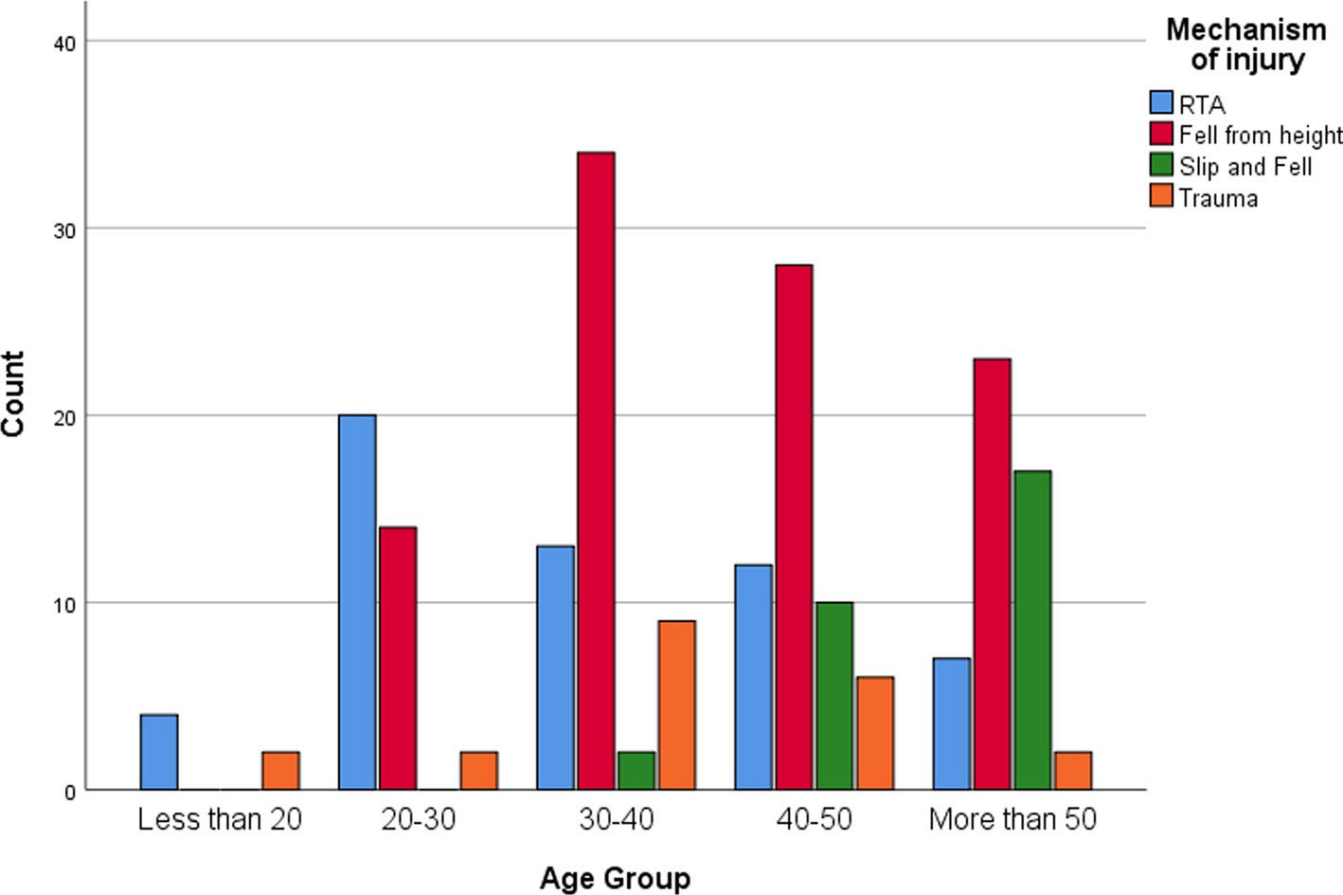
Mechanism of injury according to age group

The majority of the injuries were due to fall from height (48.1%) while 26.9% of them were due to road traffic accidents (RTA). Figure 3 demonstrates the correlation between the mechanism of injury and age of the patients over the 3-year period. The majority of the fractures in the age groups higher than 30 years of age was due to fall from height, whereas the majority of fractures in the age groups 20–30 and less than 20 were due to RTA.

**Table 1:** Demographic characteristics of participants

Variable	Mean	SD	Range
Age	40.07	11.76	45

Variable	Response	Frequency (*n* = 203 patients | 208 fractures)	Percentage (%)
Sex	Male	183/203	90.1
	Female	20/203	9.9
Laterality	Right	111/208	53.3
	Left	92/208	44.2
	Bilateral	5/208	2.5
Polytrauma	Yes	73/203	36.0
	No	130/203	64.0
Mechanism	Road traffic accident	56/208	26.9
	Fell from height	100/208	48.1
	Slip and fell	29/208	13.9
	Other	23/208	11.1

### Type of fractures and classifications

**Fig. 4:**
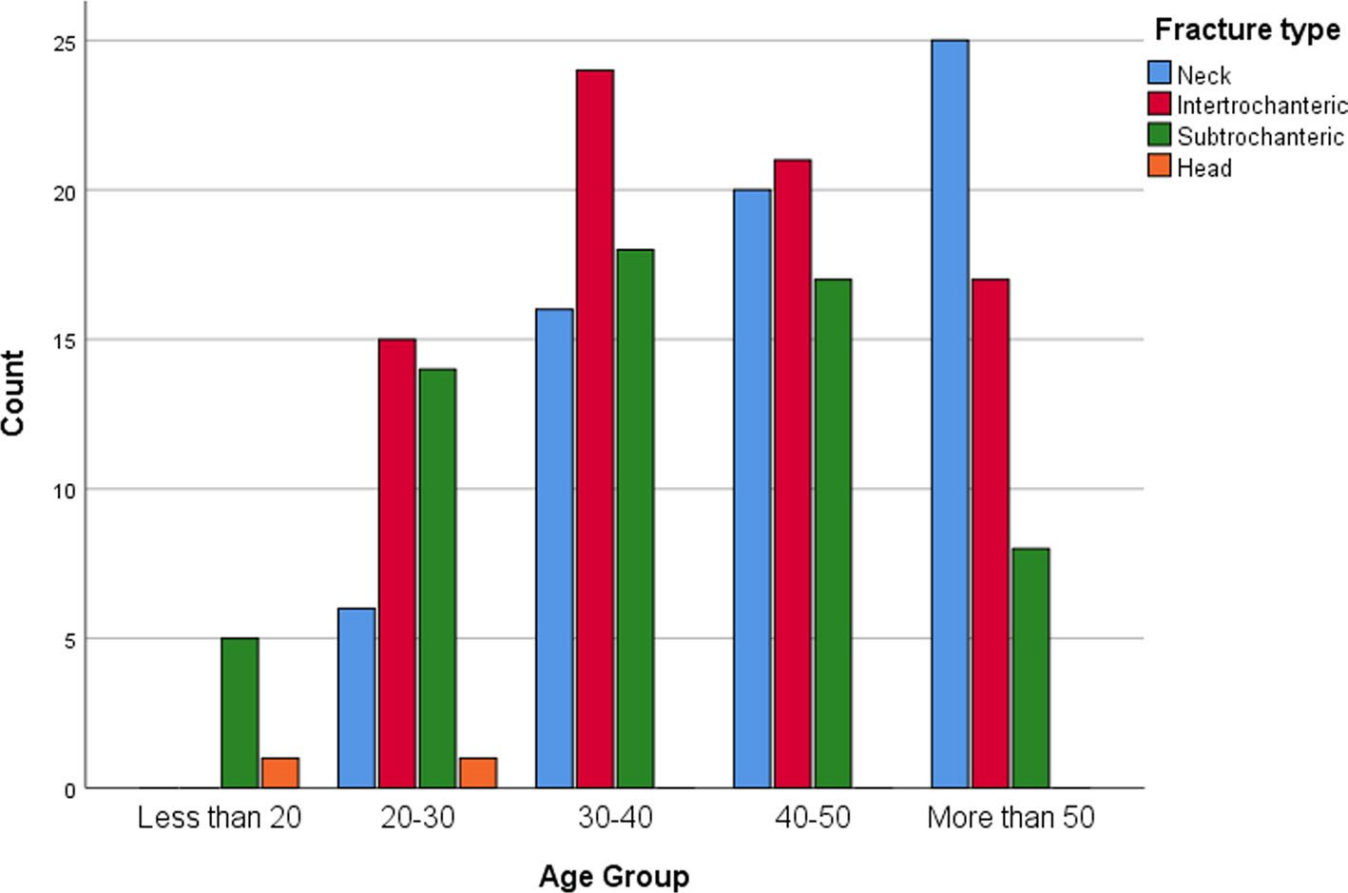
Fracture type according to age group

The most common type of proximal femoral fractures was intertrochanteric (36.1%) followed by femoral neck fractures (33.7%). Most of the fractures in the age groups 20–30, 30–40, and 40–50 were intertrochanteric fractures while the majority of the fractures in the age group ≥ 50 were femoral neck fractures (Fig. 4). Most of the fractures were classified as B2 (37.1%) followed by class A1 (22.6%) as per the AO/OTA classification. Additionally, 68.6% of the patients with femoral neck fractures were classified as P3 fractures and 30.1% of them were classified as G3 fractures (Table 2).

**Table 2:** Fractures characteristics

Variable	Response	Frequency (*n* = 203 patients | 208 fractures)	Percentage (%)
Fracture type	Complex	NOF + Shaft 1/208	2.4
		NOF + Subtrochanteric 1/208	
		Intertrochanteric + Shaft 3/208	
	Neck	68/208	32.7
	Intertrochanteric	75/208	36.1
	Subtrochanteric	58/208	27.9
	Head of femur	2/208	1.0
AO/OTA classification	A1	47	22.6
	A2	18	8.7
	A3	27	13.0
	B1	12	5.8
	B2	77	37.1
	B3	17	8.2
	C1	2	1.0
	C3	7	3.4
	Unclassifiable	1	0.5
Femur neck fracture classification	*Pauwels angle*		
	P1	9/68	13.2
	P2	13/68	19.1
	P3	46/68	67.6
	*Garden classification*		
	G1	11/68	16.2
	G2	20/68	29.4
	G3	21/68	30.1
	G4	16/68	23.5
Open fracture	Yes	5/208	2.4

### Treatment options

All patients were treated operatively. The most commonly utilized implant was caphalomedullary nail (46.7%) followed by cannulated screws (23%) and dynamic hips screw (22.5%). Only 2 patients with femoral neck fractures underwent uncemented hemiarthroplasty. The two cases of femoral head fractures were treated operatively with open reduction and screws fixation. Table 3 demonstrates a summary of treatment options.

**Table 3:** Treatment options

Implant	Number of fractures (%)
Cannulated screws	48 (23)
Cephalomedullary nail	97 (46.7)
Dynamic hip screws	47 (22.6)
Intrameduallary nail	8 (4)
Locking plate	4 (2)
Hemiarthroplasty	2 (1)
Other	2 (1)
Total	208

### Associated injuries, complications and length of hospital stay

The frequency of complications was 18.3% and the frequency of reoperations was 10.3%. On the other hand, there were no mortalities in 2017, 2018 and 2019. The mean length of hospital stays and follow up period were 10.30 ± 21.54 and 990.0 ± 128.38 days, respectively (Table 4).

**Table 4:** Complications, reoperation and mortality

Variable	Response	Frequency	Percentage (%)
Complications	Yes	38	18.3
	No	170	81.7
Reoperation	Yes	21	10.3
	No	173	85.2
Mortality	Yes	0	0.0
	No	203	100.0

## Discussion

Our review demonstrated a total incidence rate of 3.12 proximal femur fractures per 100,000 population per year between 2017 and 2019, with the majority of fractures occurring in male patients and those between 30 and 40 years of age, predominantly due to falls from height. Whereas proximal femur fractures have been previously reported to affect women more than men globally, particularly in the older age group, our results showed the opposite to be true in those younger than 60 years of age. As previously noted, more than 70% of the population of Qatar is made up of migrant male workers who fall within this age group. A technical report conducted by the Gulf Research Centre surveying the demography, migration, and labour market of Qatar in 2014 found the average age of expatriate male labourers to be 34 years [12]. Another paper aiming to outline a socio-economic profile of migrants in Qatar identified a median age of 31 years [13]. It is likely that these factors have influenced the proportion of patient characteristics described above.

The leading mechanism of injury, identified as falls from height above the age of 30 and road traffic accidents below the age of 30, provides insight into the likely circumstances under which patients are exposed to risk. The prevalence of falls from height in the working population, most of which may be work-related injuries occurring at building sites, highlights the importance of ensuring and enforcing on-site occupational safety standards. Similarly, the high prevalence of road traffic accidents in younger people under the age of 30 has been well-documented. Specifically in Qatar, young drivers in the age group 25–34 have been shown to experience the highest rate RTAs, most of whom are male drivers [14]. Road safety awareness campaigns directed toward this group of drivers may show to be useful in decreasing the incidence of fractures within this age group in the long term.

The incidence of PFF is highly variable across the world depending on the region and ethnicity studied. The highest rates have been reported in Sweden and North America, with lower rates in European countries by seven-fold, and even lower across Asia [15]. However, it is projected that more than half of all hip fractures will occur in Asia by the year 2050 [16]. Table 5 shows the incidence rates reported in previous literature from different regions of the world. Notably, most reports studied the incidence of PFF in patients aged 50 and above, a group in which osteoporosis may lead to increasingly rising rates of fracture with the dominant risk factor being older age [41], and many of these studies excluded high-energy mechanism injuries in the younger age group. There are very few previous reports aiming to identify PFF incidence rates among the non-elderly adult population. One study conducted in the Netherlands and Sweden of 1115 proximal femur fractures in 1989–1990 found only 2% of patients under 50 years of age with proximal femur fractures, which were attributed to severe trauma [42]. Thus, it is difficult to ascertain whether proximal femur fractures occur at a greater incidence among the young age group in the State of Qatar as compared with other regions of the world.

**Table 5:** The incidence rates of proximal femur fracture in different regions of the world

Place and time of study [references]	Age range studied	Incidence rate (per 100,000 person-years)		Female:Male ratio
		Male	Female	
*Middle East*				
Iran: 2003 [17]	≥ 50	115.2	115.6	1.0
Iran, Kermanshah: 2007–2008 [18]	≥ 50	181.1	214.6	1.2
Kuwait: 1992–1995 [19]	≥ 50	200	295	1.5
Kuwait: 2009–2012 [20]	≥ 50	113.7–147.4	135.3–148.1	1.3–1.5
Lebanon: 2006–2008 [21]	≥ 50	88–106	164–188	1.6–2.1
Saudi Arabia: 2017–2018 [22]	≥ 45	56.8	77.5	1.4
*Asia*				
China, Shenyang: 1994 [23]	> 50	81.0	67.0	0.8
Hong Kong: 1997–1998 [24]	≥ 50	180.0	459.0	2.6
Japan, Tottori: 1992–1994 [25]	≥ 35	57.1	145.2	2.5
Japan, Niigata: 2010 [26]	≥ 50	126.3	410.7	3.3
Malaysia: 1997–1998 [24]	≥ 50	88.0	218.0	2.5
Pakistan: 2015–2019 [27]	> 25	127.3	164.6	1.3
Thailand: 1997–1998 [24]	≥ 50	114.0	269.0	2.4
Singapore: 1997–1998 [24]	≥ 50	164.0	442.0	2.7
*Africa*				
Morocco, Rabat: 2002 [28]	≥ 50	43.7	52.1	1.2
Morocco, Rabat: 2006–2009 [29]	≥ 50	85.9	72.7	0.8
South Africa: 2017–2018 [30]	≥ 40	76·5	176·0	2.3
*Europe*				
Finland: 2013 [31]	80–84	142.5	291.9	2.0
	85–89	281.7	572.2	2.0
	≥ 90	503.2	812.5	1.6
Germany: 1985 [32]	60–64	62.1	81.7	1.3
	65–69	76.3	132.5	1.7
	70–74	103.9	233.8	2.3
	75–79	174.2	415.0	2.4
	80–84	297.9	719.9	2.4
	85 +	611.9	1252.4	2.0
Norway: 1996–1997 [33]	≥ 50	44.0	118.0	2.7
Poland: 2005 [34]	≥ 50	89.0	165.0	1.9
Portugal: 2005–2013 [35]	≥ 65	419.1	762.9	1.8
Sweden: 2015‒2018 [36]	≥ 16	123.2	237.1	1.9
Spain, Barcelona: 1984 [37]	≥ 45	115.6	252.2	2.2
*North America*				
US, Olmsted County: 1980–1989 [38]	0- ≥ 85	23.0	66.0	2.9
*South America*				
Argentina, Rosario: 2001–2002 [39]	≥ 50	137.0	290.0	2.1
Argentina, La Plata: 1989–1990 [40]	≥ 50	101.0	379.4	3.8

Amongst our studied population, the most common pattern of proximal femur fractures was intertrochanteric (36.1%), followed by femoral neck fractures (33.7%). Similar findings have been reported in previous literature [43–45]. Intertrochanteric fractures were most observed in patients younger than 50 years of age, whereas femoral neck fractures were observed in those older than 50. The opposite is true in previous literature. A study of 737 patients conducted in Baltimore between 1984 and 1986 found intertrochanteric fracture patients were older than those with femoral neck fractures [46]. In New York, a study of 717 patients between 1987 and 1994 found patients with intertrochanteric fractures to be older than femoral neck fracture patients [47]. Similarly, a study of 248 patients in Istanbul Turkey in 2008 found intertrochanteric fracture patients were significantly older than femoral neck fracture patients [48]. Another retrospective study found lower bone mineral density values among patients with intertrochanteric fractures when compared to femoral neck fractures [49]. However, the majority of the patients included in previous studies were of the elderly population with higher rates of osteoporosis presenting after simple falls. The inclusion of younger patients and high-energy mechanism injuries in our study may have given rise to this variation.

### Limitations

It is important to acknowledge that data was collected in a retrospective manner which might have introduced selection bias. In addition, the accuracy of the data is reliant upon the quality of documentation. Furthermore, as many patients return to their home country following the completion of their treatment, it was not possible to gather long-term follow up data and identify long-term complications.

## Conclusion

This study provides the initial insights into the proximal femur fractures in the young population of Qatar, shedding light on their distinct characteristics. This is the first study to investigate of the epidemiology of such fractures in this particular patient group. Remarkably, Contrary to the existing literature on older age groups, the majority of the injuries were observed in males. Falls from height followed by road traffic accidents were the primary mechanisms leading to these fractures. Improved understanding of the profile of these injuries can aid in their prevention by implementing more effective safety measures.

## Data Availability

Not applicable.

## References

[1] Lu Y, Uppal H (2019). Hip fractures: relevant anatomy, classification, and biomechanics of fracture and fixation. Geriatr Orthop Surg Rehabil.

[2] Cummings SR, Kelsey JL, Nevitt MC, O'Dowd KJ (1985). Epidemiology of osteoporosis and osteoporotic fractures. Epidemiol Rev.

[3] Maalouf G, Bachour F, Hlais S, Maalouf NM, Yazbeck P, Yaghi Y, Yaghi K, El Hage R, Issa M (2013). Epidemiology of hip fractures in Lebanon: a nationwide survey. Orthop Traumatol Surg Res.

[4] Egol KA, Leucht P (2018). Proximal femur fractures: an evidence-based approach to evaluation and management.

[5] Roberts KC, Brox WT, Jevsevar DS, Sevarino K (2015). Management of hip fractures in the elderly. J Am Acad Orthop Surg.

[6] Cooper C (1997). The crippling consequences of fractures and their impact on quality of life. Am J Med.

[7] Ahmed M, Abuodeh Y, Alhammoud A, Salameh M, Hasan K, Ahmed G (2018). Epidemiology of acetabular fractures in Qatar. Int Orthop.

[8] Population ages 15–64 (% of total population), World Bank Open Data. Available at: https://data.worldbank.org/indicator/SP.POP.1564.TO.ZS

[9] Meinberg EG, Agel J, Roberts CS, Karam MD, Kellam JF (2018). Fracture and dislocation classification compendium—2018. J Orthop Trauma.

[10] Pauwels F (1935) Der Schenkenholsbruck, em mechanisches Problem. Grundlagen des Heilungsvorganges. Prognose und Kausale Therapie. Stuttgart, Beilageheft zur Zeitschrift fur Orthopaedische Chirugie, Ferdinand Enke

[11] Van Embden D, Rhemrev SJ, Genelin F, Meylaerts SA, Roukema GR (2012). The reliability of a simplified garden classification for intracapsular hip fractures. Orthop Traumatol Surg Res.

[12] De Bel-Air F (2014) Demography, migration, and labour market in Qatar. Gulf Research Center. Available at: https://cadmus.eui.eu/bitstream/handle/1814/32431/GLMM_ExpNote_08%E2%80%932014.pdf?sequence=1

[13] Seshan G (2012). Migrants in Qatar: a socio-economic profile. J Arab Stud.

[14] Bener A (2012). A study on road traffic crashes and injuries in Qatar as reported by drivers. J Egypt Public Health Assoc.

[15] Dhanwal DK, Dennison EM, Harvey NC, Cooper C (2011). Epidemiology of hip fracture: worldwide geographic variation. Indian J Orthop.

[16] Cooper C, Campion G, Melton LJ (1992). Hip fractures in the elderly: a world-wide projection. Osteoporos Int.

[17] Moayyeri A, Soltani A, Larijani B (2006). Epidemiology of hip fracture in Iran: results from the Iranian multicenter study on accidental injuries. Osteoporos Int.

[18] Beyranvand M, Mohammadi G (2009). Incidence of hip fracture in Kermanshah, Iran. Arch Osteoporos.

[19] Memon A, Pospula WM, Tantawy QY, Abdul-Ghafar S, Suresha A, Al-Rowaih A (1998). Incidence of hip fracture in Kuwait. Int J Epidemiol.

[20] Azizieh FY (2015). Incidence of hip fracture in Kuwait: a national registry-based study. Arch Osteoporos.

[21] Sibai AM, Nasser W, Ammar W (2011). Hip fracture incidence in Lebanon: a national registry-based study with reference to standardized rates worldwide. Osteoporos Int.

[22] Saleh YAL, Sulimani RA, Alomary S (2022). Incidence of hip fracture in Saudi Arabia and the development of a FRAX model. Arch Osteoporos.

[23] Yan L, Zhou B, Prentice A, Wang X, Golden MHN (1999). Epidemiological study of hip fracture in Shenyang People’s Republic of China. Bone.

[24] Lau E, Lee J, Suriwongpaisal P (2001). The Incidence of hip fracture in four Asian countries: the Asian osteoporosis study (AOS). Osteoporos Int.

[25] Hagino H, Yamamoto K, Ohshiro H, Nakamura T, Kishimoto H, Nose T (1999). Changing incidence of hip distal radius, and proximal humerus fractures in Tottori prefecture, Japan. Bone.

[26] Miyasaka D, Endo N, Endo E (2016). Incidence of hip fracture in Niigata, Japan in 2004 and 2010 and the long-term trends from 1985 to 2010. J Bone Miner Metab.

[27] Lakho MT, Bughio S, Khan A (2022). The incidence of hip fractures in the study population: a retrospective study. Int J Curr Res Rev.

[28] El Maghraoui A, Koumba BA, Jroundi I (2005). Epidemiology of hip fractures in 2002 in Rabat, Morocco. Osteoporos Int.

[29] El Maghraoui A, Ngbanda AR, Bensaoud N (2013). Age-adjusted incidence rates of hip fractures between 2006 and 2009 in Rabat, Morocco. Osteoporos Int.

[30] Dela SS, Paruk F, Brown SL, Lukhele M, Kalla AA, Jordaan JD, Conradie M, Mohamed O, Chutterpaul P, Cassim B (2020). Ethnic and gender-specific incidence rates for hip fractures in South Africa: a multi-centre study. Bone.

[31] Kannus P, Parkkari J, Niemi S (2015). Low-trauma pelvic fractures in elderly Finns in 1970–2013. Calcif Tissue Int.

[32] Wildner M, Casper W, Bergmann K (1999). A secular trend in hip fracture incidence in East Germany. Osteoporos Int.

[33] Lofthus CM, Osnes EK, Falch JA, Kaastad TS, Kristiansen IS, Nordsletten L, Stensvold I, Meyer HE (2001). Epidemiology of hip fractures in Oslo Norway. Bone.

[34] Czerwinski E, Kanis JA, Trybulec B (2009). The incidence and risk of hip fracture in Poland. Osteoporos Int.

[35] Silva J, Linhares D, Ferreira M (2018). Epidemiological trends of proximal femoral fractures in the elderly population in Portugal. Acta Med Port.

[36] Bergh C, Wennergren D, Möller M, Brisby H (2020). Fracture incidence in adults in relation to age and gender: a study of 27,169 fractures in the Swedish fracture register in a well-defined catchment area. PLoS ONE.

[37] Diez A, Puig J, Martinez MT (1989). Epidemiology of fractures of the proximal femur associated with osteoporosis in Barcelona, Spain. Calcif Tissue Int.

[38] Pang K, Ekeuku SO, Chin K-Y (2021). Particulate air pollution and osteoporosis: a systematic review. Risk Manag Healthc Policy.

[39] Morosano M, Masoni A, Sánchez A (2005). Incidence of hip fractures in the city of Rosario, Argentina. Osteoporos Int.

[40] Bagur A, Mautalen C, Rubin Z (1994). Epidemiology of hip fractures in an urban population of Central Argentina. Osteoporosis Int.

[41] Hedlund R, Lindgren U (1987). Trauma type, age, and gender as determinants of hip fracture. J Orthop Res.

[42] Berglund-Rödén M, Swierstra BA, Wingstrand H, Thorngren KG (1994). Prospective comparison of hip fracture treatment: 856 cases followed for 4 months in the Netherlands and Sweden. Acta Orthop Scand.

[43] Douša P, Čech O, Weissinger M, Džupa V (2013). Trochanteric femoral fractures. Acta Chir Orthop Traumatol Cech.

[44] Frisch NB, Wessell N, Charters M (2018). Hip fracture mortality: differences between intertrochanteric and femoral neck fractures. J Surg Orthop Adv Spring.

[45] Huff S, Henningsen J, Schneider A, Hijji F, Froehle A, Krishnamurthy A (2022). Differences between intertrochanteric and femoral neck fractures in resuscitative status and mortality rates. Orthop Traumatol Surg Res.

[46] Fox K, Cummings S, Williams E (2000). Femoral neck and intertrochanteric fractures have different risk factors: a prospective study. Osteoporos Int.

[47] Koval K, Aharonoff G, Rokito A, Lyon T, Zuckerman J (1996). Patients with femoral neck and intertrochanteric fractures: are they the same?. Clin Orthop Relat Res.

[48] Kesmezacar H, Ayhan E, Unlu MC, Seker A, Karaca S (2010). Predictors of mortality in elderly patients with an intertrochanteric or a femoral neck fracture. J Trauma: Inj Infect Crit Care.

[49] Cho Y, Lee I, Ha SH (2020). Comparison of hip subregion bone mineral density to the type of proximal femur fracture. Arch Osteoporos.

